# Supplementary material for: The Biological Function of Genome Organization
Source: Int J Mol Sci. 2025 Sep 17;26(18):9058. doi: 10.3390/ijms26189058 (PMC12470977; doi:10.3390/ijms26189058)
Supplement: Supplementary file 1 [file ijms-26-09058-s001.zip › ijms-3818634-supplementary.pdf]

Supplementary Table S1. Acronyms and their corresponding full names

Acronym	Full name
3D	Three-dimensional
TAD	Topologically associating domain
CHIA-PET	Chromatin interaction analysis by paired-end tag sequencing
E-P	Enhancer-promoter
ChIP-seq	Chromatin immunoprecipitation sequencing
SMC	Structural maintenance of chromosome
BET	Bromodomain and extra terminal
ESC	Embryonic stem cell
LMC	Loop maintenance complex
TF	Transcription factor
FRET	Förster resonance energy transfer
FCCS	Fluorescence cross-correlation spectroscopy
PIC	Pre-initiation complex
SE	Super-enhancer
PRC	Polycomb repressive complex
HAT	Histone acetyltransferase
lncRNA	Long non-coding RNA
SNV	Single nucleotide variant
DSB	Double-strand break
TE	Trophectoderm
XCI	X chromosome inactivation
ASD	Autism spectrum disorder
ATAC-seq	Assay for transposase-accessible chromatin using sequencing
TNBC	Triple-negative breast cancer
TNM	Tumor node metastasis
CNV	Copy number variation
MM	Multiple myeloma
AML	Acute myeloid leukemia
AI	Artificial intelligence
